# High-throughput phenotyping of seminal root traits in wheat

**DOI:** 10.1186/s13007-015-0055-9

**Published:** 2015-03-01

**Authors:** Cecile AI Richard, Lee T Hickey, Susan Fletcher, Raeleen Jennings, Karine Chenu, Jack T Christopher

**Affiliations:** The University of Queensland, QAAFI, St Lucia, QLD 4072 Australia; Department of Agriculture, Fisheries and Forestry, Leslie Research Facility, Toowoomba, QLD 4350 Australia; The University of Queensland, QAAFI, 203 Tor Street, Toowoomba, QLD 4350 Australia; The University of Queensland, QAAFI, Leslie Research Facility, Toowoomba, QLD 4350 Australia

**Keywords:** Wheat breeding, Root angle, Root number, Adaptation, Drought

## Abstract

**Background:**

Water availability is a major limiting factor for wheat (*Triticum aestivum* L.) production in rain-fed agricultural systems worldwide. Root system architecture has important functional implications for the timing and extent of soil water extraction, yet selection for root architectural traits in breeding programs has been limited by a lack of suitable phenotyping methods. The aim of this research was to develop low-cost high-throughput phenotyping methods to facilitate selection for desirable root architectural traits. Here, we report two methods, one using clear pots and the other using growth pouches, to assess the angle and the number of seminal roots in wheat seedlings– two proxy traits associated with the root architecture of mature wheat plants.

**Results:**

Both methods revealed genetic variation for seminal root angle and number in the panel of 24 wheat cultivars. The clear pot method provided higher heritability and higher genetic correlations across experiments compared to the growth pouch method. In addition, the clear pot method was more efficient – requiring less time, space, and labour compared to the growth pouch method. Therefore the clear pot method was considered the most suitable for large-scale and high-throughput screening of seedling root characteristics in crop improvement programs.

**Conclusions:**

The clear-pot method could be easily integrated in breeding programs targeting drought tolerance to rapidly enrich breeding populations with desirable alleles. For instance, selection for narrow root angle and high number of seminal roots could lead to deeper root systems with higher branching at depth. Such root characteristics are highly desirable in wheat to cope with anticipated future climate conditions, particularly where crops rely heavily on stored soil moisture at depth, including some Australian, Indian, South American, and African cropping regions.

## Background

Drought is a major limiting factor of wheat (*Triticum aestivum* L.) production world-wide [1]. Water deficit during critical periods of crop development such as grain filling, can greatly impact yield stability and productivity in rain-fed agricultural systems. Traditional wheat breeding relies heavily on selection for yield per se and has contributed to significant increases in yield. However, the rate of genetic progress has slowed in recent years [2]. Yield is a quantitative trait under complex genetic control, characterized by low heritability and high genotype by environment (G × E) interactions, particularly in drought environments [3]. Physiological approaches based on proxy traits, can offer higher heritability and lower G × E interactions than selection for yield itself and complement traditional breeding approaches to accelerate improvement in drought-prone environments.

Drought-adaptive traits related to root physiology and morphology have been identified in maize (*Zea mays*), sorghum (*Sorghum bicolor*), rice (*Oryza sativa*) and wheat [4-11]. Modelling studies performed using historical climate data for wheat grown throughout the Australian cropping region indicated that root architecture had significant functional implications for the timing and amount of subsoil water extraction [4,7,12]. Wheat cultivars with narrower lateral root distribution and higher proportion of roots at depth can access more soil moisture deep in the soil profile, particularly late in the season when marginal water-use efficiency for grain production is high [4,13-17]. Such root characteristics that facilitate improved access to soil moisture late in the season are highly desirable in rain-fed systems, particularly where crops rely heavily on stored soil moisture at depth, as in parts of some Australian, Indian, South American, and African cropping regions.

Two types of roots occur in wheat, the seminal roots coming directly from the embryo and the later, nodal roots emerging at the lower tiller nodes [18]. A more vertical angle of the seminal roots and a higher number of seminal roots in wheat seedlings have been linked to a more compact root system with more roots at depth in wheat [14,19-21]. Narrow root angle and a higher number of seminal roots are considered proxy traits for selection at early growth stages in wheat breeding programs [14,15,22,23]. The association between root angle and deeper rooting systems has been demonstrated in sorghum, maize and rice, and a number of quantitative trait loci (QTL) showing homology across species have been reported recently [9,24,25].

Despite rapid advances in genomic approaches to tackle complex traits [26,27], the lack of high-throughput and large-scale phenotyping methods for root traits remains a major bottleneck to elucidate the genetic control and enable selection for such traits in breeding programs. Both field- and laboratory-based methods for phenotyping root traits have been developed [28], including soil sampling [22,29,30], thermography [6,31], X-ray computed tomography [32-36], mini-rhizotrons [37-39], rhizotrons [40,41], and non-soil techniques [14,21,42-44]. However, most of these approaches are low-throughput. Laboratory-based methods can be limited in their ability to reproduce field-like conditions [45-47]. For example, soil-environment × genotype interactions significantly affect the root length of wheat cultivars grown in sandy soil compared to agar plates [48]. Yet, root studies performed in the laboratory are generally less laborious and less time-consuming than in the field, and can be conducted out-of-season. In addition, root measurements tend to be more precise and more reproducible because the plants are grown in a more homogeneous environment compared to the field.

In this study, we used a panel of 24 spring wheat cultivars to design and evaluate two high-throughput methods for measuring seminal root angle and number in controlled environment growth facilities, one based on clear pots and the other based on growth pouches. We discuss the advantages and disadvantages of these root trait phenotyping methods, along with the opportunity to exploit high-throughput phenotypic screening in breeding populations.

## Results

### Genetic variation for seminal root angle and number

**Figure 1:**
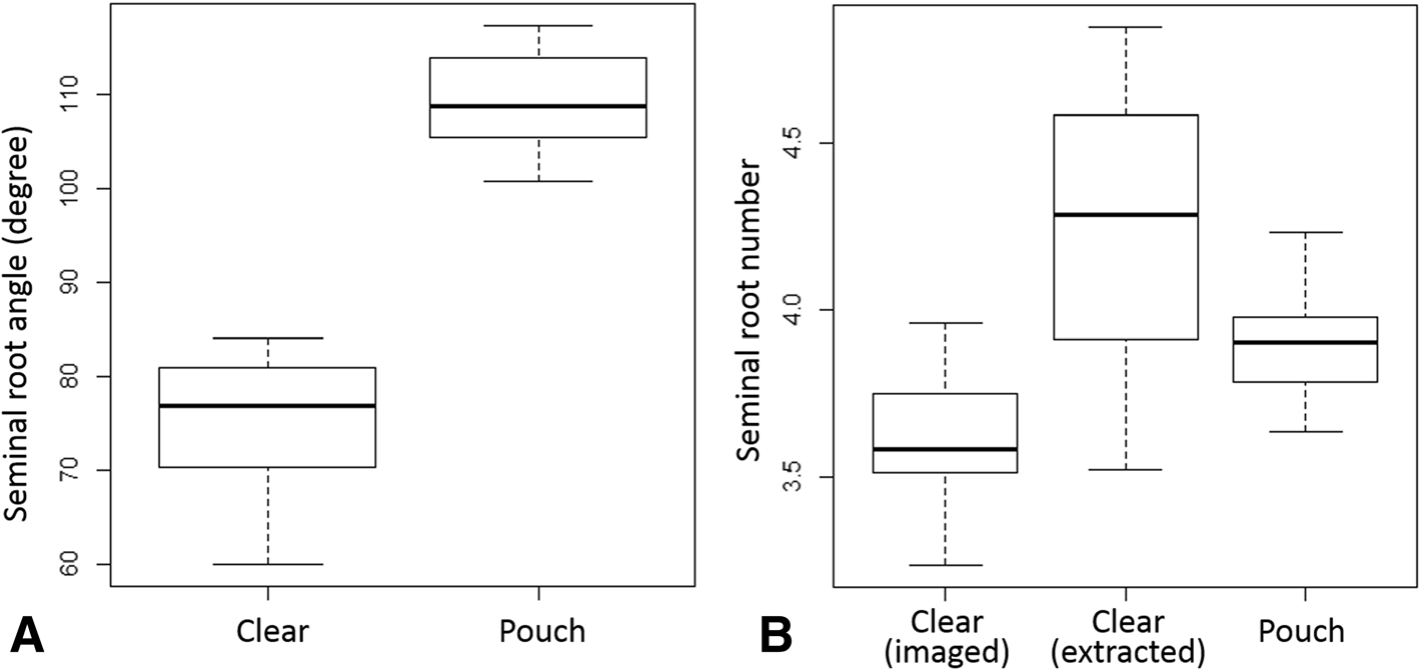
**Genetic variation for seminal root angle and number.** Box and whisker plots of **(A)** seminal root angle and **(B)** seminal root number, for the panel of 24 wheat cultivars evaluated using the clear pot and growth pouch methods. The values correspond to the average BLUPs per cultivar of the two clear pot experiments Clear_1 and Clear_2 (Clear) and the two growth pouch experiments Pouch_1 and Pouch_2 (Pouch). The seminal root number for the clear pot method was measured either via image analysis (imaged) or by counting roots after removing seedlings from soil (extracted). The bottom and the top of the boxes display the first and third quartile values for each experiment, respectively. The band inside the box displays the median and the ends of the whiskers display the minimum and maximum values.

In the clear pots, seedling roots grew along the wall and were clearly distinguished from the dark soil. At the time of imaging for seminal root angle (i.e. five days after sowing), the first pair of seminal roots had elongated on each side of the radicle, with an average seminal root angle of 75.5° for the two clear pot experiments. By contrast, in the growth pouches, seedling roots grew freely in the air space between the moistened paper and the plastic. At the time of scanning (i.e. 20 days after sowing), first and often second pairs of seminal roots had elongated on each side of the radicle, however, only the angle between the first pair was considered here. The average seminal root angle across the two pouch experiments was 109.7°. The observed range in seminal root angle phenotypes varied between methods, the clear pot method provided a range in seminal root angle from 60.1 to 84.0°, while the growth pouch method produced a wider seminal root angle with a range from 100.8 to 117.4° (Figure 1A).

Seminal root number was measured six days later than seminal root angle in the clear pot experiments (i.e. at 11 days after sowing). In both clear pot experiments, the root number estimated non-destructively from the images was significantly lower (p-value < 0.001) compared to measures obtained by extracting the seedlings from the soil; average across the two experiments was 3.6 for imaged and 4.2 for extracted, respectively. In the pouch experiments, seminal root number was measured at the same time as root angle (i.e. at 20 days after sowing) and seedlings exhibited 3.9 roots on average across the experiments. The genotypic range in seminal root number phenotypes varied between methods, with the clear pot method providing the widest range in seminal root number (3.2–4.0 for imaged and 3.5–4.8 for extracted) compared to the growth pouch method (3.6–4.2) (Figure 1B).

### Comparison of methods

The heritability for seminal root angle was higher for the clear pot method (h^2^ = 0.65) compared to the growth pouch method (h^2^ = 0.52) (Table 1). However, the heritability for each individual experiment displayed some variability within methods, with higher values for Clear_1 and Pouch_2 (h^2^ = 0.79 and h^2^ = 0.63, respectively) compared to Clear_2 and Pouch_1 (h^2^ = 0.51 and h^2^ = 0.42, respectively) (Table 1). For seminal root number, the heritability was the highest for the clear pot method, with higher heritability obtained for extracted root number (h^2^ = 0.80) compared to imaged root number (h^2^ = 0.50) (Table 1). The heritability for seminal root number was the lowest for the growth pouch method (h^2^ = 0.37) (Table 1). Overall, the heritability for each individual experiment was quite consistent within methods (Table 1).

**Table 1:** **Statistics for the seminal root angle and number**

			**Heritability (h** ^**2**^ **)**	**Genetic variance**	**Error variance**	**Observations per cultivar**
**Seminal root angle**	Clear	Clear_1	0.79	39%	61%	6.2/10
		Clear_2	0.51	16%	84%	5.7/10
		**Clear average**	**0.65**	**28%**	**72%**	**6.0/10**
	Pouch	Pouch_1	0.42	6%	55%	4.5/6
		Pouch_2	0.63	14%	78%	5.3/6
		**Pouch average**	**0.52**	**10%**	**67%**	**4.9/6**
**Seminal root number**	Clear (imaged)	Clear_1	0.45	9%	91%	8.2/10
		Clear_2	0.54	12%	86%	8.8/10
		**Clear average**	**0.50**	**10%**	**90%**	**8.5/10**
	Clear (extracted)	Clear_1	0.80	33%	66%	8.2/10
		Clear_2	0.79	30%	69%	8.8/10
		**Clear average**	**0.80**	**32%**	**68%**	**8.5/10**
	Pouch	Pouch_1	0.37	9%	88%	5.2/6
		Pouch_2	0.36	8%	70%	5.7/6
		**Pouch average**	**0.37**	**9%**	**79%**	**5.5/6**

Heritability h^2^, genetic variance, error variance and average number of observations for seminal root angle and number for the panel of 24 wheat cultivars evaluated using different methods based on clear pots and growth pouches. The values correspond to the individual experiments. The values in bold correspond to the average of the two clear pot experiments Clear_1 and Clear_2 (‘Clear average’) and the two growth pouch experiments Pouch_1 and Pouch_2 (‘Pouch average’). The seminal root number for the clear pot method was measured in two different ways: based on images (imaged) and after extracting the seedlings (extracted).

The error variance was higher than the genetic variance for all experiments (Table 1), indicating that there were more differences in the seminal root angle and number within cultivar individuals than across cultivar averages. Almost all variation was explained by the genetic and error variance in the clear pot experiments. However, the random factors “Pouch” and “Box” had a significant effect in the growth pouch experiments.

The clear pot experiments (Clear_1 and Clear_2) used 10 reps per cultivar (i.e. 240 seeds in total per experiment), while the growth pouch experiments (Pouch_1 and Pouch_2) used only 6 reps per cultivar (i.e. 144 seeds in total per experiment). The number of observations for each experiment varied between experiments, as in both methods some seeds didn’t germinate and some roots were too short (<3 cm) to measure the seminal root angle. Using the clear pot method, some roots were also hidden by the soil on the images, making measurement impossible. Roots were sometimes hidden by the soil close to the surface, but visible deeper down, making the root angle measurement impossible but the imaged root number possible. In contrast, in the growth pouch method roots were always visible when present. The average number of observations per cultivar for seminal root angle was 6.0 (out of 10) for the clear pot experiments and 4.9 (out of 6) for the growth pouch experiments (Table 1). For seminal root number, the average number of observations per cultivar were 8.5 (out of 10) for the clear pot method for both imaged and extracted seminal root number, while for the pouch method, observations were obtained for 5.5 (out of 6) plants per cultivar (Table 1).

**Figure 2:**
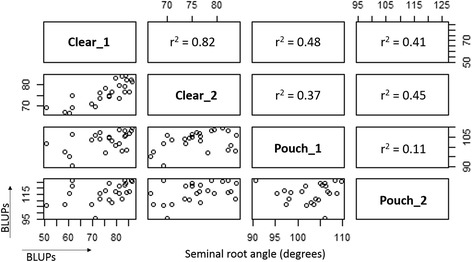
**Genetic correlations of seminal root angle using clear pot and growth pouch methods.** Genetic correlations (upper panels) and scatter plots (lower panels) of the BLUPs for seminal root angle (in degree) between the clear pot (i.e. Clear_1 and Clear_2) and the growth pouch (i.e. Pouch_1 and Pouch_2) experiments. Data represents average BLUPs of the 24 wheat cultivars.

The genetic correlations for the seminal root angle were the highest between the two clear pot experiments Clear_1 and Clear_2 (r^2^ = 0.82) and the lowest between the two growth pouch experiments Pouch_1 and Pouch_2 (r^2^ = 0.11) (Figure 2). The ranking of cultivars for root angle was almost the same across the two clear pot experiments, but differed markedly between the two growth pouch experiments. For instance, the cultivar Chara was the narrowest in Pouch_1, but one of the widest in Pouch_2 (data not shown). The genetic correlation between the two methods, clear pot and growth pouch, were medium (r^2^ ranging 0.37–0.48) (Figure 2).

**Figure 3:**
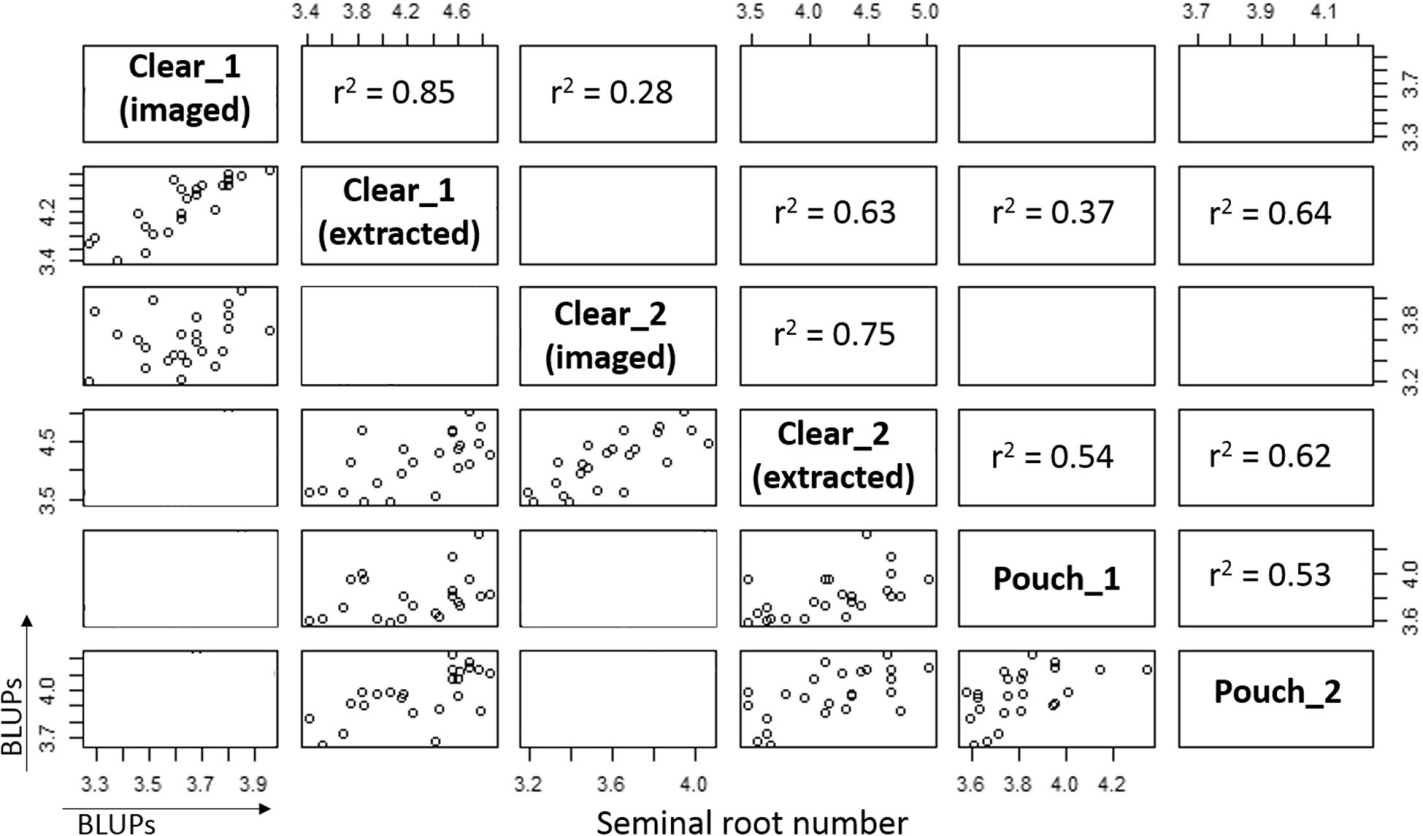
**Genetic correlations of seminal root number using clear pot and growth pouch methods.** Genetic correlations (upper panels) and scatter plots (lower panels) of the BLUPs for seminal root number counted based on images (imaged) and after extracting the seedlings (extracted) for each of the clear pot experiments (i.e. Clear_1 and Clear_2), and for the seminal root number with the growth pouch experiments (Pouch_1, and Pouch_2). Data represents average BLUPs of the 24 wheat cultivars.

Genetic correlations between imaged and extracted seminal root number were high for both Clear_1 and Clear_2 experiments (r^2^ = 0.85 and 0.75, respectively; Figure 3). The genetic correlations were high between the two clear pot experiments (Clear_1 and Clear_2) for the extracted seminal root number (r^2^ = 0.63), but low for the imaged root number (r^2^ = 0.28) (Figure 3). For the growth pouch method, the genetic correlation between the two experiments (Pouch_1 and Pouch_2) was medium (r^2^ = 0.53), as well as the genetic correlations between clear pot (extracted) and growth pouch methods (r^2^ ranging 0.37–0.64) (Figure 3). There was no significant genetic correlation between the seminal root angle and number for both the clear pot and growth pouch experiments (data not shown).

### Diversity for root angle in Australian wheat cultivars

**Figure 4:**
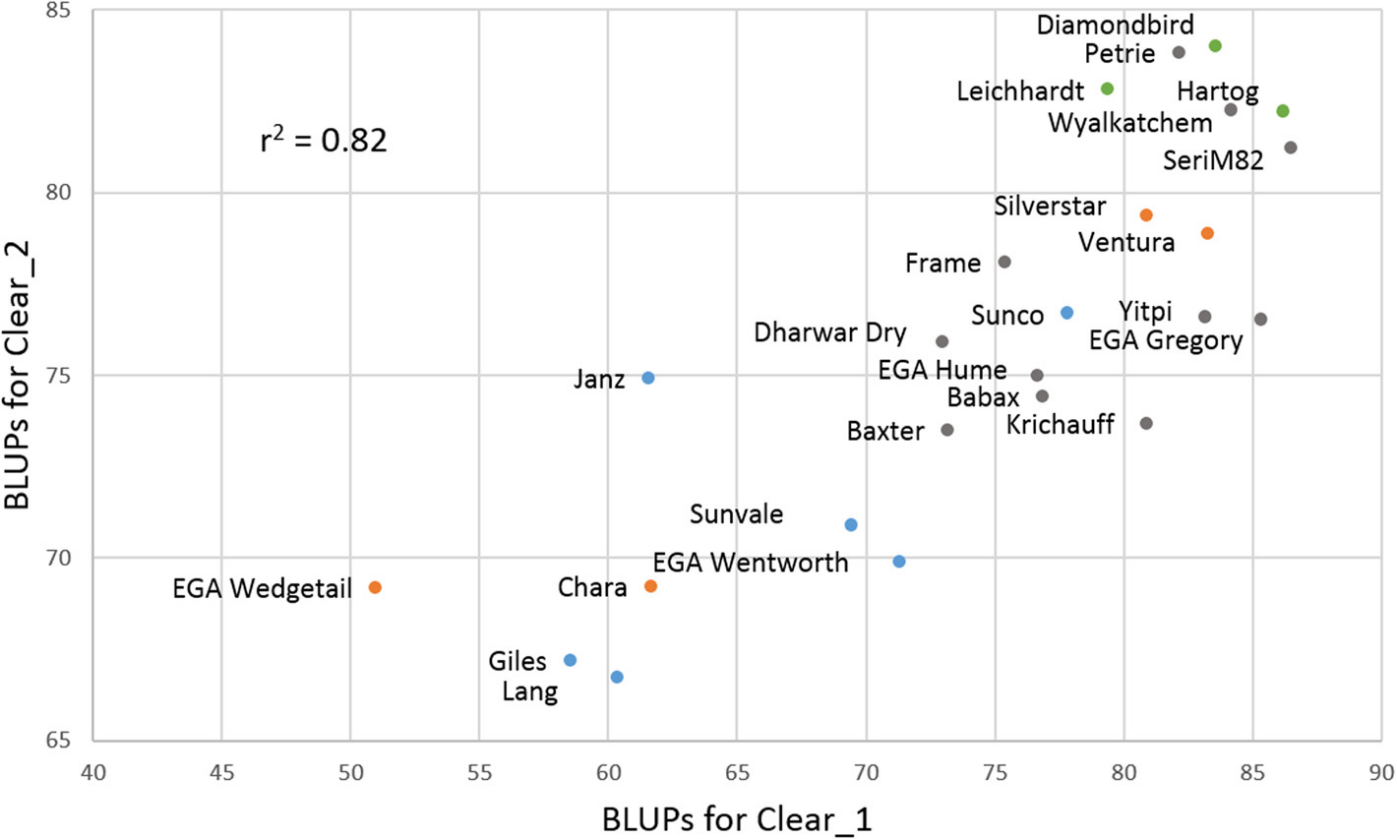
**Seminal root angle of the panel of 24 wheat cultivars.** Scatter plot of BLUPs for seminal root angle (in degrees) between the two clear pot experiments (i.e. Clear_1 and Clear_2) for 24 wheat cultivars. Blue dots = Cook-type, green dots = Pavon-type, orange dots = Cook/Pavon type, grey dots = other backgrounds.

The cultivar ranking for seminal root angle was almost the same across the two clear pot experiments (Figure 4). Some trends based on genetic backgrounds could be observed, with all the Cook-type cultivars (EGA Wentworth, Giles, Janz, Lang, Sunco, and Sunvale) having narrower roots than all the Pavon-type cultivars (Diamonbird, Hartog, and Leichhardt) (Figure 4). The Cook/Pavon-type cultivars (Chara, EGA Edgetail, Silverstar, and Ventura) displayed a mixture of narrow and wide seminal root angle phenotypes as might be anticipated. Cultivars belonging to other genetic backgrounds did not show a consistent pattern of seminal root angle.

## Discussion

The two phenotypic methods for seminal root traits evaluated in this study permitted differentiation of seminal root angle and number in the panel of 24 wheat cultivars. The clear pot method showed consistency across experiments and is considered the most suitable for large-scale and high-throughput screening of seedling root characteristics in crop improvement programs.

In this study, we examined the seminal root angle and number for a panel of 24 wheat cultivars measured using two methods; one based on clear pots and the other using growth pouches. The clear pot method provided a higher degree of variation for both seminal root traits with a range of 23.9° for root angle and 1.3 for extracted root number. This compared to the growth pouch method with a range of 16.6° and 0.6 roots per plant. It should be noted that these ranges may not represent the full extent of genetic variation in wheat germplasm, as this panel represents a limited set of genotypes and many share similar pedigrees and/or genetic backgrounds. Higher levels of variation for these traits were observed for the same 24 wheat cultivars in a previous study (72.4–112.6° i.e. 40.2° for root angle and for root number 3.2–5.0 i.e. 1.8 roots per plant) using a gel chamber method [14]. However, this method is labour intensive and not suitable for evaluation of large numbers of entries.

Despite variations within experiments, the heritability was higher using the clear pot method for both seminal root traits (i.e. h^2^ = 0.65 for root angle and h^2^ = 0.80 for extracted root number) compared to the growth pouch method (h^2^ = 0.52 for root angle and h^2^ = 0.37 for root number). If implemented in breeding programs, the relatively high heritability should enable genetic gain for this trait. The achieved number of observations for seminal root angle using the clear pot method was lower than the potential 10 observations due to the fact that some roots were hidden by soil in the images. As a consequence, this method requires a high number of repetitions (i.e. ~10) to ensure high heritability. The position of the seed at sowing (i.e. embryo pointed downwards and slightly towards the wall) is critical to ensure roots grow along the wall and are visible. The achieved number of observations for seminal root traits using the growth pouch method was close to the potential 6 observations due to the fact that roots were always visible when present. The heritability could be improved by increasing the number of reps, for example 10 reps instead of 6. The error variance was higher than the genetic variance for all experiments, which is not surprising considering that traits were measured for single plants. Results from the two clear pot experiments were more strongly correlated (r^2^ = 0.82 for root angle and r^2^ = 0.63 for extracted root number), when compared to results from the two growth pouch experiments (r^2^ = 0.11 for root angle and r^2^ = 0.53 for root number). The rank of the cultivars based on the seminal root angle and number was quite consistent across the two clear pot experiments, suggesting that the method is repeatable and has power to detect differences in root phenotypes (i.e. narrow/wide seminal root angle, low/high number of seminal roots). The wider range of root phenotypes obtained using the clear pot method enabled better differentiation among cultivars with more repeatable results, and thus appears superior to the growth pouch method for implementation in breeding programs.

Seminal root number was measured with the clear pot method in two different ways: by counting based on images and after seedlings were extracted from the soil. Roots were underestimated using the images because some roots were hidden by soil, resulting in a significantly lower average number of seminal roots for the imaged root number compared to the extracted root number. As expected, the extracted root number was more accurate than the imaged root number. For instance, the genetic variation, the heritability and the genetic correlations were higher for the extracted seminal root number than the imaged values. However, imaged and extracted seminal root number were strongly correlated (r^2^ > 0.75) and ranking of cultivars using both techniques was also very similar. Despite a lower level of precision, estimation of seminal root number using the imaging technique is preferred for breeding purposes because this method doesn’t require a labour intensive transplanting of the selected plants. For instance, the imaging method can be used to differentiate extreme phenotypes (i.e. low versus high root number), in order to enrich segregating populations with desirable genes via repetitive cycles of selection. However, to precisely phenotype or characterize fixed lines, counting the roots after pulling out the plants may be preferred.

The paper growth media in growth pouches and the agar gel of the gel-filled chamber method from Manschadi et al. [14] both provide conditions less representative of natural soils than the soil-based growth medium used in the clear pot system. Consequently the soil-based clear pot method may result in phenotypes more similar to those expressed in the field [48]. In addition, the growth pouch and gel-filled methods are very time-consuming and labour intensive to set up, thus, are better suited for evaluation of smaller numbers of cultivars compared to the clear pot method. For these reasons, we propose that the clear pot method is preferred for high-throughput and large-scale screening of seminal root angle and number.

The rank between cultivars based on the seminal root angle calculated with the clear pot method was almost identical across the two experiments and ranking seemed to correspond with the genetic background of the wheat cultivars. For instance, most of the cook-type cultivars displayed a narrow seminal root angle, while all the Pavon-type cultivars displayed wider seminal root angles, which is similar to previous studies [14,23]. The Cook-type cultivars tend to have a longer season maturity compared to the Pavon-type cultivars used in this study. Cultivars with a longer cycle are more likely to encounter terminal moisture stress in the season, particularly if grown in a summer dominant rainfall environment. Deeper rooting could be an adaptation for late cultivars to ensure photosynthetic and remobilization activities during grain filling in rainfed wheat production systems relying heavily on deep stored soil moisture. There was little consistency between the preferred growing region for the Australian wheat cultivars evaluated in this study and cluster analysis based on root angle phenotypes also failed to detect any obvious trends other than those associated with genetic background (data not presented). Although wheat breeders have likely indirectly selected for desirable root architecture where environmental pressure is frequent, this is not the only trait affecting drought adaptation. In fact, while drought types highly differ depending on the season and region [49-51], drought adaptation typically involves the interaction of a number of traits related to water utilization as well as other physiological processes [2,52]. As a result, breeders and pre-breeders are targeting other traits such as adapted phenology [53], transpiration efficiency [54], cooler canopy temperature [16,55,56] and reduce tillering [57]. While deep root architecture is likely important for adaptation in rainfed wheat production systems relying heavily on stored soil moisture (particularly at depth [4]), this trait may be less advantageous in other environments, for example where rainfall is more frequent through the growing season, where soils are compacted [58] or for late sown conditions [59].

Selection for combinations of physiological traits that underpin yield may be a more effective way to achieve genetic gain for yield in specific environment types, rather than direct selection for yield per se [3,51,60,61]. The clear pot method allows high-throughput and cost-effective screening of breeding populations at a rate of 600 plants/m^2^ in a controlled environment within only five days for the seminal root angle and 11 days for the seminal root number. The technique is suitable for characterising both fixed lines and for screening large segregating populations (e.g. F2 and F3). As the system permits growing-on of the selected plants, repeated cycles of selection can be performed across consecutive generations to rapidly enrich breeding populations with desirable alleles for root traits. Alternatively, the method could be used to select parental lines with desired root traits for crossing. Therefore, the clear pot method has the potential to accelerate genetic gain for drought tolerance in breeding programs*.*

The technique is also well adapted for use in the “speed breeding” system developed and refined at The University of Queensland that achieves rapid plant growth by incorporating controlled temperature and constant light [62]. By combining speed-breeding growth conditions and the root trait phenotypic screening method, it is possible to achieve up to 30 phenotypic screens within 12 months if plants are not grown to maturity. Alternatively, under optimised growth conditions, up to 6 consecutive cycles of selection could be achieved in 12 months with selections grown through to maturity producing seed in each generation. Thus, within a 12 month timeframe, it would be possible to make crosses, screen and produce seeds for F1 to F4 generations for desirable root traits, and produce F5:F6 lines with improved root traits. Also, seminal root trait screening can be easily integrated with other phenotypic screening methods adapted to the speed breeding system, such as adult plant resistance to rust pathogens [63] and grain dormancy for tolerance to pre-harvest sprouting [64]. We anticipate this methodology will accelerate identification of genetic diversity for root traits in wheat and propose that it could be applied to other crops, such as barley and chickpea.

## Conclusions

Phenotyping root traits in wheat has been limited by the availability of suitable methods. In this study, we reported a new high-throughput method using clear pots to phenotype seminal root angle in 5-day-old wheat seedlings and seminal root number in 11-day-old wheat seedlings. This method has clear advantages over other previously reported techniques and could be easily integrated into wheat breeding programs targeting drought tolerance via improved access to deep soil water.

## Methods

A panel of wheat cultivars differing for their region of adaptation and drought tolerance were assayed in clear pots and growth pouches for seminal root angle and number. In total, four experiments were conducted in this study – two based on clear pots (i.e. Clear_1 and Clear_2) and two based on growth pouches (i.e. Pouch_1 and Pouch_2) to assess the robustness and repeatability of each method.

### Clear pot method

Two experiments using the clear pot method (‘Clear_1’, and ‘Clear_2’) were conducted successively under the same conditions to evaluate the panel of 24 wheat cultivars for seminal root angle and seminal root number.

**Figure 5:**
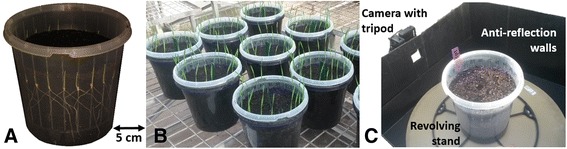
**Wheat seedlings phenotyped for seminal root traits in a high-throughput system using clear pots. (A)** Wheat seedlings grown in clear pots under controlled environment conditions (picture taken five days after sowing). **(B)** The clear pots placed inside black pots to exclude light (picture taken at 11 days after sowing). **(C)** Images recorded for each plant of each pot using a camera fixed on a tripod, a black box with anti-reflection walls and a revolving stand.

Wheat seedlings were cultured in 4 L clear pots (ANOVApot®, 200 mm diameter, 190 mm height, http://www.anovapot.com/php/anovapot.php). The clear pots were filled with a pine bark potting media (70% composted pine bark 0–5 mm, 30% coco peat, pH 6.35, EC = 650 ppm, nitrate = 0, ammonia < 6 ppm and phosphorus = 50 ppm). Seeds were sown at a depth of 2 cm every 2.5 cm along the pot wall, providing a density of 24 seeds per pot (600 plants/m^2^). The seeds were carefully placed vertically, embryo downwards and facing the wall to facilitate root growth along the transparent wall (Figure 5A). After sowing, the clear pots were placed inside 4 L black pots (ANOVApot®, 200 mm diameter, 190 mm height) to exclude light from the developing roots (Figure 5B). The pots were watered after sowing and no additional water or nutrients were supplied thereafter.

The two experiments used randomised complete block designs where 24 cultivars were randomised across 10 pots, ensuring cultivars were present only once in each pot. Each pot represented one replicate block and one plant of each cultivar in each pot represented the experimental unit.

The two experiments were conducted in a walk-in, temperature-controlled growth facility. Constant temperature (17°C ± 2 C) was adopted over 24 hours with diurnal (12 hour) natural light.

**Figure 6:**
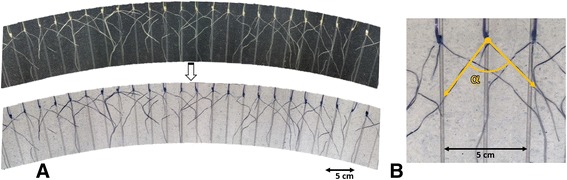
**Measuring seminal root angle with the clear pot method. (A)** Panoramic image of wheat seedling grown in the clear pot system obtained by stitching images of individual plants using software (*PhotoStitch*) and colours inverted to facilitate root identification. **(B)** For each plant, the angle (α) between the first pair of seminal roots was measured at approximately 3 cm distance from the seed using software (*ImageJ*).

Five days after sowing, images of the seminal roots visible through the clear wall were recorded using a camera (Canon PowerShot SX600 HS 16MP Ultra-Zoom Digital Camera) fixed on a tripod (Slik F153 Tripod) (Figure 5C). Images were recorded for each plant by rotating the pot 15° in a clockwise direction. The images captured from each pot displayed some overlap and were joined together to create a panoramic image for the whole pot with the stitching software *PhotoStitch* (http://support-au.canon.com.au/contents/AU/EN/0200246607.html) (Figure 6A). This step reduced the picture file storage size and also improved image analysis speed by using 1 picture per pot instead of 24. Colours of panoramic images were inverted to enhance the contrast between roots and soil, facilitating root-trait measurements with the software *imageJ* (http://imagej.nih.gov/ij/) [65] (Figure 6A).

For each plant, the growth angle between the first pair of seminal roots was measured at approximately 3 cm distance from the seed (Figure 6B).

In this study, we tested two different ways to measure seminal root number at 11 days after sowing. The “imaged” number of seminal roots was measured based on the photographic images by counting the number of roots emerging from the seed. The “extracted” number of seminal roots was measured after pulling out the wheat seedlings and counting the number of roots.

### Growth pouch method

Two experiments (‘Pouch_1’ and ‘Pouch_2’) were conducted successively under the same conditions to evaluate the panel of 24 wheat cultivars for seminal root angle and seminal root number using the growth pouch method.

**Figure 7:**
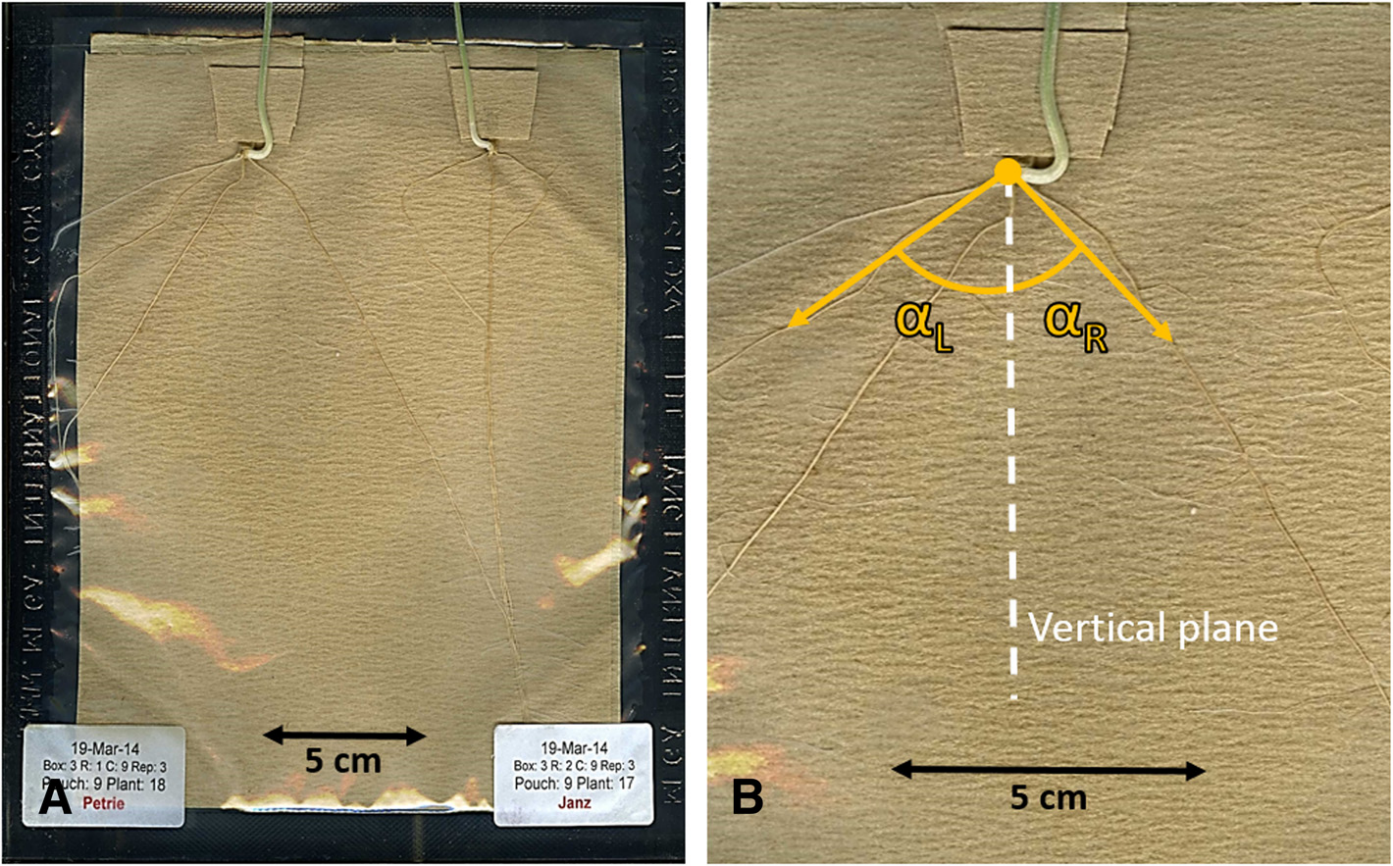
**Illustration of a growth pouch. (A)** Wheat seedlings were phenotyped for seminal root angle and number using growth pouches (picture taken 20 days after sowing). **(B)** For each plant, the left (α_L_) and the right (α_R_) angle between each of the first pair of seminal roots and the vertical plane was measured at approximately 3 cm distance from the seed using software (*Opengelphoto*).

The experiments were performed using Cyg germination growth pouches (Mega International, http://www.mega-international.com/index.htm). Measuring 18 cm × 16.5 cm, the plastic pouches contained perforated germination paper that has been folded to form a continuous trough along the top of the pouch, in which seeds are supported (Figure 7A). To avoid roots spatially interfering with each other during the initial growth period, each pouch contained only two seeds (Figure 7A). Pouches were pre-prepared by removing excess paper from the seed trough, leaving two individual troughs (Figure 7A). Tap water (15 mL) was added to each pouch and allowed to evenly distribute over the germination paper. Dry seeds were placed vertically into the troughs, with the embryo end pointing down, and the embryo facing out towards the plastic. Pouches were then placed vertically into containers, sandwiched between foam to maintain even pressure on the seeds and to reduce air spaces. Containers were covered in cling wrap to prevent moisture loss.

Pouches were placed into a plant growth cabinet at a constant temperature of 15°C with no light. After 12 days, lights were turned on using a 12 h photoperiod. Seedlings were grown for 20 days in total.

The pouch experiments used a resolvable block design where pouches constituted a block size of 2. This ensured pairs of cultivars were not in the same pouch together more than once. Each experiment had 6 boxes with 16 pouches in each box set out in a 2 × 8 array. Each box comprised a replicate block, with 1 replicate of the panel of 24 cultivars, 1 extra replicate for Hartog and SeriM82, and 1 replicate of 6 other cultivars. The randomisation for the pouch experiments were latinised.

Seminal root angle and number were measured using a scanner (Epson Perfection 4990 Photo) at 20 days after sowing. The images were analysed using a specifically-designed software program *Opengelphoto*, which enables measurement of angle of individual roots from a vertical plane. For each seedling the growth angle between each of the first pair of seminal roots (i.e. left and right first pair of seminal roots) and the vertical plane was measured at approximately 3 cm distance from the seed (Figure 7B). The root number was measured by counting the number of roots based on the scanned images.

### Statistical analysis

A linear mixed model framework was used to analyse genotype-environment interactions across experiments based on clear pots (Clear_1 and Clear_2) and growth pouches (Pouch_1 and Pouch_2). The mixed model contained random components that identified the structure of the experimental design for each experiment: (i) Pot for the clear pot experiments, and (ii) Pouch and Box for the growth pouch experiment. Given the importance of genotype ranking across experiments, the random model formula also included Genotype as a random effect. The random model formula allows for estimation of variance heterogeneity for each of the random terms for each experiment. The residual maximum likelihood (REML) algorithm [66] was used to provide estimates of the variance components and the best linear unbiased predictions (BLUPs). Data were analysed with ASReml-R [67] using R software Version 3.0.0 (R Core team 2013).

For seminal root angle measured using the growth pouch method, each plant had two values corresponding to the angle between the left or right seminal roots and the vertical plane. Therefore, the dataset for seminal root angle measured using the growth pouch method had an additional factor Side (left and right). After the analysis, the BLUPs were multiplied by two to allow comparison with the seminal angle measured using the clear pot method. For seminal root number, a Student test was performed to compare the means between imaged and extracted root number using R software Version 3.0.0.

### Plant material

The study was conducted using a panel of 24 spring wheat cultivars (Table 2), that was previously characterized for seminal root angle and root number using a gel-filled chamber method reported by Manschadi et al. [14]. In their study, Manschadi et al. [14] obtained seminal root angles ranging from 36.2° to 56.3° and number of seminal roots ranging from 3.2 to 5.0. These seminal root angle values corresponded to the angle between each of the seminal roots and the vertical plane and were multiplied by two to allow comparison with the seminal angle measured in this study.

**Table 2:** **Name, origin and genetic background of the 24 wheat cultivars used in this study**

**Cultivar**	**Breeding program** ^**1**^	**Genetic background**
Babax	CIMMYT	Veery
Baxter	QDPI	CIMMYT/Cook
Chara	DPI Vic	Cook/Pavon
Dharwar Dry	Central India	CIMMYT
Diamondbird	NSW DPI	Pavon
EGA Gregory	EGA	Pelsart/Batavia
EGA Hume	EGA	Pelsart/Batavia
EGA Wedgetail	EGA	Cook/Pavon
EGA Wentworth	EGA	Cook
Frame	AGT	Condor/Gabo
Giles	QDPI	Cook
Hartog	QDPI	Pavon
Janz	QDPI	Cook
Krichauff	AGT	Condor/Gabo
Lang	QDPI	Cook
Leichhardt	QDPI	Pavon
Petrie	QDPI	Pelsart/Batavia
SeriM82	CIMMYT	CIMMYT/Veery
Silverstar	NSW DPI	Cook/Pavon
Sunco	Uni Syd	Cook
Sunvale	Uni Syd	Cook
Ventura	NSW DPI	Cook/Pavon
Wyalkatchem	AgWA	Condor/Gabo
Yitpi	AGT	Condor/Gabo

^1^Breeding program abbreviations: Queensland Department of Primary Industries (QDPI), Department of Primary Industries Victoria (DPI Vic), Australian Grain Technologies (AGT), New South Wales Department of Primary Industry (NSW DPI), International Maize and Wheat Improvement Center (CIMMYT), Enterprise Grains Australia (EGA), Western Australia Department of Agriculture (AgWA), University of Sydney (Uni Syd).

The panel comprised 21 Australian spring wheat cultivars, including some of the most widely grown throughout Australia in recent years, two elite cultivars (Babax and SeriM82) from the International Maize and Wheat Improvement Center (CIMMYT) in Mexico and one wheat cultivar from India (Dharwar dry).
